# Incidence and predictors of chronic kidney diseases among type 2 diabetes mellitus patients at St. Paul’s Hospital, Addis Ababa, Ethiopia

**DOI:** 10.1186/s13104-018-3618-9

**Published:** 2018-07-31

**Authors:** Alemayehu Hussen Geletu, Alemayehu Shimeka Teferra, Malede Mequanent Sisay, Destaw Fetene Teshome

**Affiliations:** 1 Department of Reproductive Health, School of Public Health, College of Medicine and Health Sciences, Addis Ababa University, Addis Ababa, Ethiopia; 2 Department of Epidemiology and Biostatistics, Institute of Public Health, College of Medicine and Health Sciences, University of Gondar, 196, Gondar, Ethiopia

**Keywords:** Chronic kidney diseases, Type 2 diabetes, Diabetic complications, Predictors

## Abstract

**Objective:**

This study aimed to estimate the incidence of chronic kidney disease and its predictors among newly diagnosed type 2 diabetes patients attending St. Paul’s Hospital, Addis Ababa, Ethiopia.

**Results:**

The overall incidence of chronic kidney disease was a major public health issue among type 2 diabetes mellitus patients with 2178 (95% CI 12,801, 21,286) cases per 10,000 patient-months. Moreover, 62(14.25%) patients in the sample experienced chronic kidney disease. Old age [adjusted hazard ratio (AHR) = 1.06, 95%CI 1.03, 1.09], no diabetic retinopathy [AHR = 0.13, 95%CI 0.07–0.24], high density lipoprotein cholesterol ≥ 40 mg/dl [AHR = 0.55, 95%CI 0.31, 0.97] and high body mass index [AHR = 1.17, 95%CI 1.1, 1.25] were common factors for chronic kidney diseases.

**Electronic supplementary material:**

The online version of this article (10.1186/s13104-018-3618-9) contains supplementary material, which is available to authorized users.

## Introduction

Chronic kidney disease contributes substantially to human suffering and death [1]. For instance, the estimated prevalence of people who have experienced CKD is 11–13% and 13.9% worldwide and sub-Saharan Africa respectively. chronic kidney disease is a critical issue in the management of patients with type 2 diabetes mellitus [1–5].

Previous studies in the area indicated that is mainly driven by diabetic mellitus (DM). For example, type 2 diabetes mellitus (T2DM) causes 30–50% of CKD cases [6–10]. According to recent literature, CKD is more prevalent (11–29%) are among type 2 diabetic patients in developed than in developing countries [11–13]. A similar h study done in Hong Kong and South Africa reported a development of CKD among type 2 DM patients at a rate of 12.7 and 94.9%, respectively [14, 15]. Although the prevalence of CKD ranged from 2 to 41% in Africa [5, 16], the growing burden of CKD among type 2 DM patients is not well explored in low and middle-income countries [17–20].

Several studies highlighted the risk factors for CKD complication of type 2 diabetic patients. For example, age and sex were significant risk factors for CKD in type 2 diabetic patients [21–23]. However, studies found disparities in the risk factors for chronic kidney diseases [13, 22, 24–26]. In another cohort study, physical activity reduces the incidence [21], whereas smoking history and lipid abnormalities have increased the risk of CKD in type 2 DM patients [12, 27–31]. In another prospective study done among type 2 diabetic patients, factors such as high systolic blood pressure (SBP), low diastolic blood pressure (DBP) and high body mass index (BMI) significantly affected the CKD [12, 29, 32, 33]. Moreover, hypoglycemia, history of hypertension and diabetic retinopathy were considerable risks [5, 12, 22, 34–36]. A systematic review and meta-analysis revealed that BP lowering medications and intensive blood-glucose control can reduce the risk of CKD [37, 38].

Previous studies showed that the incidence of diabetic kidney disease varied across countries. But this emerging global public health problem is not well investigated, rather it is overlooked in low-come countries including Ethiopia. Quantifying the burden of the disease and early detection of the risk factors is paramount in the prevention of CKD. Therefore, this study aimed to estimate the incidence of chronic kidney disease and its predictors among newly diagnosed type 2 diabetes patients attending St. Paul’s Hospital, Addis Ababa, Ethiopia.

## Main text

### Methods

An institution-based retrospective follow-up study was conducted at St. Paul’s Hospital, Addis Ababa, Ethiopia. St. Paul’s Referral Hospital is found in the capital of Ethiopia, Addis Ababa. Since 1969 the hospital has been providing different medical care services included chronic problems like chronic kidney disease and supports to an estimated 200,000 people annually referred from all over the country.

All newly diagnosed T2DM patients who enrolled at St. Paul’s Referral Hospital between January 2008 and November 2017 were considered in this study. The required sample (435) was determined by the incidence and predictors of CKD using STATA software. New T2DM diagnosed patients were eligible, while those who had CKD at the time of the diagnosis for T2DM were excluded from the study.

The data were collected using a standard extraction checklist which was adapted from the World Health Organization (WHO) guidelines [39]. After they were checked for completeness, data were entered using Epi Info 7 and exported to STATA 12 for further analysis. The outcome variable in this study was time to chronic kidney disease. Chronic kidney disease was defined as an estimated Glomerular Filtration Rate (eGFR) < 60 ml/min/1.73 m^2^ estimated by the Cockcroft-Gault equation. Accordingly, participants were classified as either CKD cases or censored at the end of the study. Furthermore, the incidence of CKD was determined from the start of type 2 DM diagnosis until the last follow-up visit.

We used the Weibull Cox regression models to identify the predictors. Variables in the bi-variable proportional hazard model with a P-value below 0.2 were included in the multivariable analysis. For the overall model, Scaled Schoenfeld and Cox-Snell residuals plots were used to check the assumption. The most parsimonious model was chosen by Akaike’s Information Criterion (AIC). Finally, the crude and adjusted hazard ratio (HR) with its 95% CI was calculated to determine statistical significance.

### Results

A total of 435 newly diagnosed T2DM patients were included in this study. Approximately half (50.6%) were females and only 101(23.2%) treated with insulin. More than half, 235(54%) had a history of hypertension, 404 (92.9%) had no history of diabetic retinopathy, and 261(66%) had a baseline LDL cholesterol level below 100 mg/dl. Slightly more than half (50.5%) of the patients had less than 40 mg/dl HDL cholesterol level. Similarly, 225(56.4%) had triglyceride level greater or equal to 150 mg/dl and 259(64.6%) had less than 200 mg/dl total cholesterol level [Table 1].

**Table 1:** Baseline factors of newly diagnosed T2DM patients in St. Paul’s Hospital, Addis Ababa, from January 2008 to April 2017

Variables	Categories	Frequency	Percent
Sex	Male	215	49.4
	Female	220	50.6
Types of DM therapy	Insulin	101	23.2
	Oral	334	76.8
History of hypertension	No	198	45.5
	Yes	237	54.5
Anti-hypertensive therapy	No	251	57.7
	Yes	184	42.3
History of diabetic retinopathy	No	404	92.9
	Yes	31	7.1
LDL cholesterol, mg/dl	< 100	261	66.0
	≥ 100	135	34.0
HDL cholesterol, mg/dl	< 40	200	50.5
	≥ 40	196	49.5
Total cholesterol, mg/dl	< 200	259	64.6
	≥ 200	142	35.4
Triglyceride, mg/dl	< 150	174	43.6
	≥ 150	225	56.4

The median time to develop CKD was 70.9 months with an interquartile range of (IQR: 41.00–88.73 months). The overall incidence rate of CKD was 2178 (95% CI 12,801, 21,286) cases per 10,000 patient-month with total 28,466.13 patient-months observation. Moreover, the proportion of CKD among newly diagnosed T2DM patients was 14.25%.

The Kaplan–Meir curve showed that the chance of developing CKD for a patient who has T2DM increased over time. A large number of CKD diagnosed T2DM patients were observed between 60 and 80 months of the study period [Additional file 1]. Furthermore, a comparison of the survival functions of patients who had diabetic retinopathy complications showed a shorter CKD complication time than patients who didn’t have (log-rank *X*^2^ = 67.4, P < 0.0001). Survival differences were also observed in patients who had HDL Cholesterol level (log-rank *X*^2^ = 14.07, P < 0.0001).

**Fig. 1:**
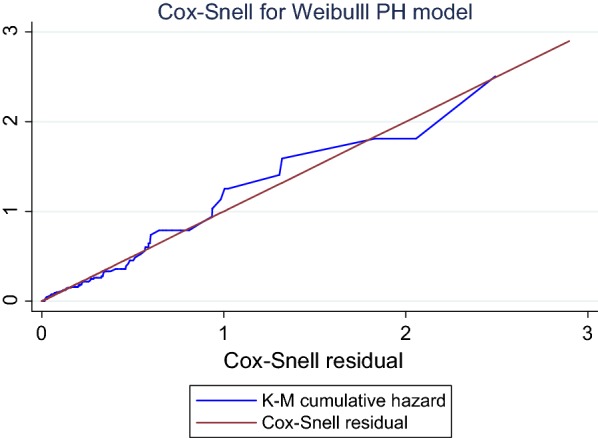
Cox-Snell residuals for Weibull PH models of newly diagnosed T2DM patients in St. Paul’s Hospital, AA, from January 2008 to April 2017

Based on AIC, Weibull Cox regression model was the most efficient model to describe the data (AIC = 243.2). According to the Schoenfeld residual global test, the overall full model satisfies the proportional hazard assumption (*X*^2^ = 6.04, P < 0.481). As well, the Cox Snell residual plot showed the proportional hazard assumption was satisfied (Fig. 1).

In multivariable, the Weibull Cox regression, age, history of diabetic retinopathy, high HDL cholesterol and BMI were significantly associated with the likelihood of CKD among type 2 diabetic patients.

The risk of developing CKD among newly diagnosed T2DM patients rose by 6% with a 1-year increase in age [AHR = 1.06, 95% CI 1.03–1.09]. The risk of developing CKD decreased by 87% [AHR = 0.13, 95% CI 0.07–0.24] among newly diagnosed T2DM with no history of diabetic retinopathy than patients with diabetic retinopathy. The risk of developing CKD decreased by 45% among newly diagnosed type 2 diabetic patients with high HDL cholesterol level (≥ 40 mg/dl) than patients with low HDL cholesterol level [AHR = 0.55, 95% CI 0.31–0.97]. Furthermore, for a unit increase in BMI, the hazard of developing CKD among newly diagnosed T2DM patients increases by 17% [AHR = 1.17, 95% CI 1.1–1.25] [Table 2].

**Table 2:** Multivariable analysis of Weibull PH model for newly diagnosed T2DM patients in St. Paul’s Hospital, AA, from January 2008 to April 2017

Variables	Category	CKD status		Crude HR [95% CI]	Adjusted HR [95% CI]
		Censored	Event		
Sex	Male	185	30	1	
	Female	188	32	0.94 (0.57, 1.54)	
Age				1.07 (1.04, 1.09)	1.06 (1.03, 1.09)*
Diabetic retinopathy	Yes	357	47	1	1
	No	16	15	0.11 (0.06, 0.2)	0.13 (0.07, 0.24)*
History of hypertension	No	174	26	1	
	Yes	199	36	1.18 (0.71, 1.95)	
Hypertensive therapy	No	213	38	1	
	Yes	160	24	0.83 (0.5, 1.4)	
Types of DM therapy	Insulin	85	16	1	
	Oral	288	46	0.96 (0.54, 1.7)	
FBG(mg/dl)				0.99 (0.995, 1.002)	
SBP (mmHg)				1.02 (1.01, 1.03)	
DBP (mmHg)				1.03 (1.01, 1.05)	
LDL cholesterol	< 100 mg/dl	221	40	1	
	≥ 100 mg/dl	113	22	1.02 (0.61, 1.72)	
HDL cholesterol	< 40 mg/dl	155	45	1	1
	≥ 40 mg/dl	179	17	0.35 (0.2, 0.62)	0.55 (0.31, 0.97)*
Total cholesterol	< 200 mg/dl	221	38	1	
	≥ 200 mg/dl	118	24	1.09 (0.65, 1.8)	
Triglyceride	< 150 mg/dl	221	38	1	
	≥ 150 mg/dl	118	24	1.35 (0.81, 2.25)	
BMI (kg/m2)				1.2 (1.1, 1.3)*	1.17 (1.1, 1.25)*
LR test χ^2^(6) = 96.7, P > χ^2^ = 0.0000, Log likelihood = − 113.6					AIC = 243.2

* P-value < 0.05

### Discussion

This study investigated the incidence and predictors of chronic kidney disease among newly diagnosed T2DM patients at St. Paul’s Hospital, Addis Ababa, Ethiopia.

In the study, the cumulative incidence of CKD was 2178 (95% CI 12,801, 21,286) cases per 10,000 patient-months. This study revealed a higher incidence of CKD than the studies done in Hong Kong [14], Italy [11], Sweden [13] and Spain [35]. The differences could be attributed to the short follow up time used by the other studies and the variations in diagnostic methods applied. Moreover, the black race was associated with a greater rate of GFR decline which increases the incidence of CKD [40]. However, it was lower than that of a study done in UK [12]. This could be due to the difference in the study design used by the U.K, which was a prospective follow up study.

In this study, we have found that age is a risk factor for CKD. The finding is consistent with those of studies conducted in U.K [12], Taiwan [21], Japan [22], and Italy [41]. This might be because getting older age is associated with such risk factors CKD as cardiovascular diseases and obesity as well as endothelial cell dysfunction, activation of the sympathetic nervous system which promotes glomerulonephritis and chronic renal failure [42, 43]. This makes the mean change in eGFR decline with increases in age [44].

Patients with high BMI were significantly associated with CKD. The finding is inconsistent with those of studies conducted in Sweden [13], Italy [30, 45] and Israel [46]. The possible reason could be that an increased BMI promotes kidney damage through direct mechanisms like hemodynamic and hormonal effects. This might also lead to glomerular hyper fusion and glomerular hyper filteration; as a result, glomerular capillary pressure increases which may subsequently result in an increased urinary albumin excretion, followed by overt proteinuria and declining GFR [47]. Weight loss intervention is assistant for medical treatments to prevent or delay the progression of CKD in overweight or obese people with type 2 diabetes [48].

This study found that the presence of diabetic retinopathy at baseline was a high risk for CKD. This finding is in line with those studies conducted in Japan [22] and UK [12]. This might be associated with the elevation of cardiac biomarkers and endothelial dysfunction which in turn lead to circulatory abnormalities increases proteinuria and reduces vascular reactivity that reflects systemic factors associated with the development of CKD [49, 50].

In this study, we found that higher levels of HDL cholesterol (≥ 40 mg/dl) were associated with a lower risk for CKD. This result was in concordance with those of studies conducted in Italy [30] and Japan [22]. Biologically, HDL cholesterol may be protective against the incidence of CKD. Moreover, type 2 diabetes patients might be primarily involved in the reverse cholesterol transport and in a number of other non-cholesterol dependent effects, including anti-oxidant, anti-inflammatory, anti-thrombotic and vasopressin effects. All of these beneficial vascular effects may also act to explain the lower risk of CKD incidence [30].

### Conclusion

In this study, the incidence of CKD among type 2 DM patients was moderately growing burdens and seek public concerns. Old age, high BMI, history of diabetic retinopathy and lower levels of HDL cholesterol were risk factors for the development of CKD. More attention and close monitoring should be exercised in order to control high BMI, high HDL cholesterol and diabetic retinopathy among the elderly.

## Limitations

This investigation attempted to estimate the incidence of CKD and identify risk factors for the disease which may serve as input for policy and program designing in the context of a developing country. Indeed, this study included only type 2 diabetic patients who attended the hospital. Selection bias might have occurred if patients lived at home undiagnosed during the time. Additionally, the research did not investigate the behavioral and institutional characteristics because they were not available in the hospital records, making it difficult to assess possible confounders. Therefore, the findings should be interpreted with this limitation in mind.

## Additional file

**Additional file 1.** Kaplan–Meier cumulative hazard estimate of newly diagnosed T2DM patients at St. Paul’s Hospital, Ethiopia.

## Abbreviations

AHR: adjusted hazard ratio
AIC: Akaike information criteria
BMI: body mass index
CKD: chronic kidney disease
DM: diabetes mellitus
HDL: high-density lipoprotein
T2DM: type 2 diabetes mellitus
WHO: World Health Organization
