# Supplementary material for: Incidence and predictors of chronic kidney diseases among type 2 diabetes mellitus patients at St. Paul’s Hospital, Addis Ababa, Ethiopia
Source: BMC Res Notes. 2018 Jul 31;11:532. doi: 10.1186/s13104-018-3618-9 (PMC6069572; doi:10.1186/s13104-018-3618-9)
Supplement: Supplementary file 1 [file 13104_2018_3618_MOESM1_ESM.docx]

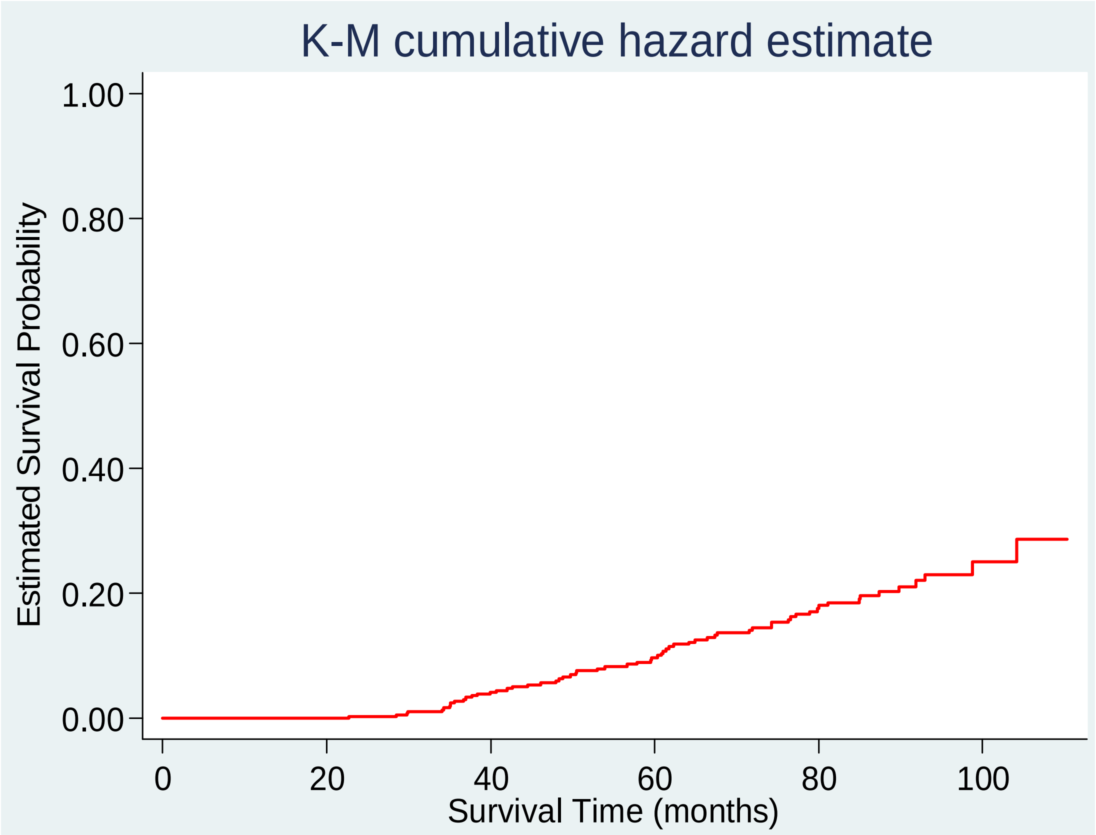
K-M cumulative hazard estimate
Estimated Survival Probability
1.00
0.80
0.60
0.40
0.20
0.00
0
20
40
60
80
100
Survival Time (months)
